# Bone mineral density as a prognostic marker in patients with biliary tract cancer undergoing surgery

**DOI:** 10.1038/s44276-024-00094-2

**Published:** 2024-09-23

**Authors:** Lisa Heinrichs, Georg Fluegen, Sven H. Loosen, Christina Loberg, Linda Wittig, Alexander Quaas, Patrick S. Plum, Nils Große Hokamp, Peter Minko, Andreas Krieg, Gerald Antoch, Wolfram T. Knoefel, Tom Luedde, Christoph Roderburg, Markus S. Jördens

**Affiliations:** 1 Department of Gastroenterology, Hepatology and Infectious Diseases, University Hospital Düsseldorf, Medical Faculty of Heinrich Heine University Düsseldorf, 40225 Düsseldorf, Germany; 2 Department of Surgery (A), Heinrich-Heine-University and University Hospital Duesseldorf, 40225 Duesseldorf, Germany; 3 Department of Diagnostic and Interventional Radiology, University Hospital Düsseldorf, Medical Faculty of Heinrich Heine University Düsseldorf, 40225 Düsseldorf, Germany; 4 Institute of Pathology, University Hospital Cologne and Medical Faculty, University of Cologne, 50937 Cologne, Germany; 5 Department of General, Visceral, Cancer and Transplantation Surgery, University Hospital Cologne, 50937 Cologne, Germany; 6 Institute for Diagnostic and Interventional Radiology, University Hospital Cologne, 50937 Cologne, Germany

## Abstract

**Background:**

Biliary tract cancer (BTC) is one of the most aggressive malignancies and surgery represents the only curative treatment approach. However, even in patients with complete tumor resection 5-year survival rates are below 30%. So far, prognostic markers to assess the outcome of these patients are lacking. We therefore evaluated bone mineral density (BMD) as a prognostic tool in patients receiving surgery for BTC.

**methods:**

76 BTC patients undergoing tumor resection in our clinic (Duesseldorf cohort) as well as an external validation cohort of 34 BTC patients (Cologne cohort) were included. BMD was analyzed at the first lumbar vertebra, using routine CT scans which has been proven comparable to DXA.

**Results:**

Median overall survival (OS) of the Duesseldorf cohort after surgery was 527 days, one- and five-year survival probabilities were 62 and 18%. Patients with BMD above 156.5 HU had significantly improved OS (1435 days vs. 459 days; *p* = 0.002). The prognostic value for BMD was confirmed using Cox-regression analysis, as well as an external validation cohort. In subgroup analysis the prognostic effect of BMD was only present in female patients, suggesting sex specific differences.

**Conclusion:**

BMD is a valuable, easily accessible and independent prognostic marker in patients receiving liver surgery for BTC.

## Introduction

The assessment of the patients´ body composition for prognostic and predictive purposes has become increasingly important in medicine [1]. Measuring body composition allows the estimation of body tissues, organs and their distribution in living individuals. The role of changes in body composition has been analyzed in manifold malignancies and sarcopenia, the most common body composition abnormality, has been identified as a prognostic and predictive factor in several types of cancer, including gastrointestinal tumors [2, 3].

Cholangiocellular carcinoma (CCA) is the second most common liver tumor with an increasing incidence over the last years. In combination with gallbladder cancer it can be referred to as biliary tract cancer (BTC). Treatment options for all entities remain limited and tumor resection is the only potentially curative therapy [4]. However, many patients already have advanced disease at the time of diagnosis and the decision whether a patient will benefit from extensive surgery is often difficult to make in routine practice. To date, few objective markers exist beyond the clinical judgment of the treating physician to stratify patients according to their presumed clinical course after surgery. We previously established two markers for sarcopenia, L3SMI and L3PMI, as prognostic markers for survival in BTC patients [5, 6]. Bone mineral density (BMD) represents another surrogate for the patients´ body composition and was suggested as a potential marker for estimating the patients´ prognosis in many diseases [7]. In clinical routine, the BMD is assessed using dual-energy X-ray absorptiometry (DXA). However, DXA is not used for staging examinations in BTC and therefore not available for clinical decision making in most patients. Nevertheless, it was recently demonstrated that BMD can be analyzed by routine CT imaging, which is available in almost all cancer patients [8, 9]. In the present study we examined the role of BMD, determined from pretherapeutic CT scans performed as part of clinical staging, as a potentially novel tool to estimate whether a patient may benefit from extended surgery for BTC.

## Material and methods

### Selection of study patients

**Fig. 1:**
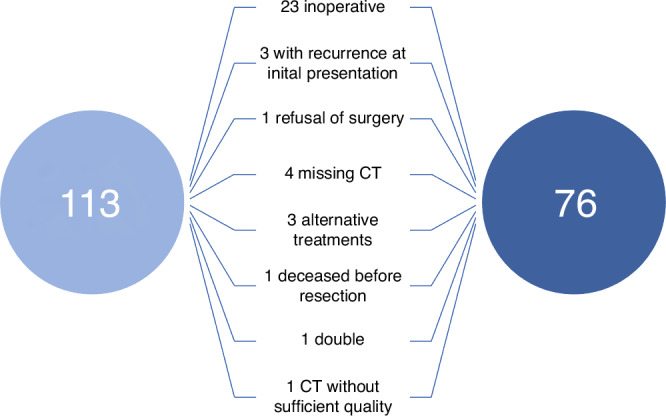
Patient selection of the Duesseldorf cohort. 37 patients had to be excluded from the initial cohort due to reasons indicated.

In total, 113 patients who underwent surgery for BTC at the Department of General, Visceral and Pediatric Surgery at the University Hospital Düsseldorf between 2011 and 2021 were screened for the study (Duesseldorf cohort). Detailed patient characteristics have recently been published and are further shown in Table 1 [5]. In total, 37 patients were excluded from the analysis due to different reasons like inoperability or poor CT scan quality (Fig. 1). For validation of our findings, we used a cohort of 34 patients treated at the University Hospital of Cologne (Cologne cohort, supplementary Table 1). The study was approved by the ethics committee of the medical faculty, Heinrich Heine university Düsseldorf (2021-1334_1).

**Table 1:** Study cohort Düsseldorf.

Parameter	Study cohort
BTC patients	*n* = 76
sex (%)	
male	48.7 (37)
female	51.3 (39)
Age (years, median and range)	68.5 (41–89)
BMI (kg/m^2^, median and range)	26.08 (15.96–44.26)
Tumor localization (%)	
iCCA	68.4 (52)
Klatskin	11.8 (9)
dCCA	3.9 (3)
gallbladder	15.8 (12)
Staging (%)	
UICC I	26.3 (20)
UICC II	28.9 (22)
UICC III	23.7 (18)
UICC IV	18.4 (14)
ECOG PS (n, %)	
0	59 (77.6)
1	9 (11.8)
2	4 (5.3)
unknown	4 (5.3)
Chemotherapy (n, %)	
neoadjuvant	4 (5.3)
adjuvant	21 (27.6)
Surgical procedure (n, %)	
Hemihepatectomy	14 (18.42)
Extended hemihepatectomy	9 (11.84)
Segment resection	35 (46.05)
Whipple / PPPD	4 (5.26)
CHE	1 (1.32)
Whipple + segment resection	3 (3.95)
Hemihepatectomy + segment resection	2 (2.63)
Bile duct resection	1 (1.32)
Other	7 (9.21)
Overall survival (days, median and range)	527 (2–3087)
Recurrence free survival at 12 months (%)	34.2% (26)
Bone mineral density L1 (HU, median and range)	130 (51.65–253)

*BTC* biliary tract cancer, *iCCA* intrahepatic cholangiocellular adenocarcinoma, *dCCA* distal cholangiocellular adenocarcinoma, *UICC* Union for International Cancer Control, *ECOG PS* Eastern Cooperative Oncology Group Performance Status.

### Analysis of bone mineral density

The local PACS (IntelliSpace PACS, Philips, Amsterdam, The Netherlands) was used for BMD analysis of routine CT scans obtained during preoperative tumor staging. The trabecular BMD of the first lumbar vertebra was measured in Hounsfield units (HU) within a manually placed region of interest (ROI), as described recently (Supplementary Fig. 1) [10]. Variations due to the venous plexus in the posterior part or to inhomogeneous trabecular structure in the middle of the vertebral body were excluded by placing the ROI in the anterior part of the upper third of the vertebral body.

### Statistical analysis

Statistical analysis was performed using SPSS 27 (SPSS, Chicago, IL, USA) as described in detail before, unless otherwise stated [11]. Correlation analysis was performed using Spearman correlation. Shapiro-Wilk test was used to assess normal distribution. Non-parametric data was compared by Mann–Whitney-U- or Kruskal-Wallis-Test. Box plots indicate medians, quartiles and ranges. The impact of different parameters on overall survival was investigated by Kaplan-Meier curve analysis. Log-rank test was used to indicate statistical differences between various groups. As described before, we used the optimal cut-off finder to assess the optimal cut-off values for bone density using method “significance of correlation with survival variable” that fits Cox proportional hazard model to the dichotomized variable and the survival variable [12]. For the analysis of the progostic value of different variables regarding overall survival we used univariate and multivariate Cox-regression analysis. 95% confidence interval (CI 95%) and hazard ratio (HR) are displayed. *P* values less than 0.05 were considered statistically significant. We used the Kaplan Meier method to estimate the one-year and 5-year survival probability using the survival package in R (v.4.2.2).

## Results

### Baseline characteristics of patients

A total of *n* = 76 patients receiving liver surgery for BTC between 2011 and 2021 were included into the training cohort (Duesseldorf cohort), as recently described [5]. We observed 51 events (deaths) among 76 patients. The median age was 68.5 years; 51.3% of patients were female. Intrahepatic cholangiocarcinoma (iCCA) was the most common localization of BTC (68.4%), while 11.8% of patients presented with Klatskin tumors, 15.8% of patients with gallbladder carcinoma and 3.9% with distal CCA (dCCA). Most patients were in good performance status (ECOG 0, 77.6%). 5.3% of patients had received neoadjuvant chemotherapy, 27.6% adjuvant chemotherapy. Median overall survival (OS) was 527 days, with a recurrence-free survival at 12 months of 34.2%. The one- and five-year survival probabilities were 62% (95% CI: 52–75%) and 18% (95% CI: 10–33%). For the external validation cohort, 34 patients were enrolled at the University Hospital Cologne (Cologne cohort). Table 1 and supplementary table 1 provide a detailed summary of clinical patient characteristics.

### Bone mineral density is dependent on age but independent of sex or tumor stage

**Fig. 2:**
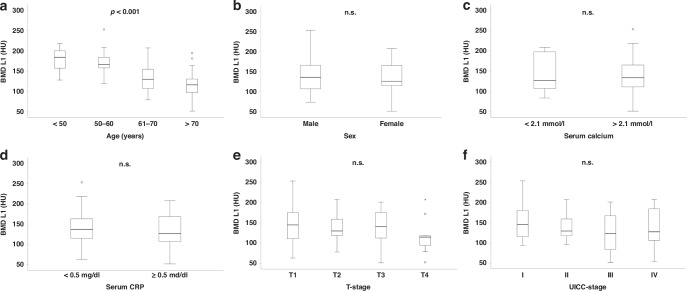
BMD dependance on various clinical or laboratory parameters. BMD was significantly decreased in older patients (**a**), but sex, serum calcium concentration, serum CRP, T- or UICC-stage had no influence on BMD (**b**–**f**). Box plots show medians, quartiles, and ranges; *p* ≤ 0.05 was considered statistically significant. Circles represent outliers, stars represent far outliers.

BMD was determined from preoperative CT scans within the trabecular space of the first lumbar vertebra. As expected, in the Duesseldorf cohort BMD was significantly reduced in older patients compared to younger ones (*p* < 0.001; Fig. 2a), which was also confirmed by Spearman correlation analysis (−0.571; *p* < 0.001). In contrast, neither the patients´ sex, nor serum calcium concentration or CRP had an influence on the individual BMD value (Fig. 2b–d). Similarly, the BMD was independent of the patients´ tumor stage (Fig. 2e, f), highlighting that BMD represents a stable parameter in patients with biliary tract cancer.

### BMD is a prognostic marker for overall survival in patients undergoing surgery for BTC

**Fig. 3:**
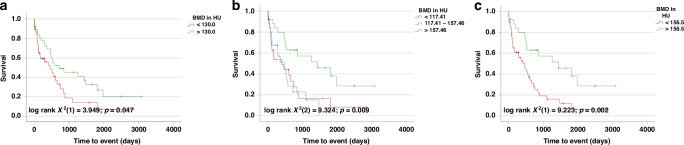
Prediction of survival after resection of BTC using BMD. Patients with BMD at level L1 above the median of 130 HU (**a**), the 66th percentile (157.46 HU) (**b**) or the optimal cut-off (156.5 HU) (**c**) had significantly improved OS.

Based on previous studies suggesting a prognostic function of BMD in patients receiving systemic treatments for BTC, we evaluated the prognostic value of BMD in patients receiving curative intended liver surgery for BTC in the Duesseldorf cohort [13]. As there is no established cut-off value for BMD in CCA patients, we subdivided our cohort into two groups with BMD levels above or below the median (130 HU). Of note, in Kaplan-Meier curve analyses, patients with BMD above 130 HU had a significantly longer OS compared to patients with a BMD below this threshold (724 (95% CI: 0–1462) vs. 459 (95% CI: 119–799) days; *p* = 0.047; Fig. 3a). As the median level of BMD might not represent the ideal cut-off value for the differentiation between survivors and non-survivors, we next used the 33rd and 66th percentile as cut-off. Strikingly, patients with BMD above 157.46 HU—representing the 66th percentile–had a significantly improved survival compared to patients below that value (>66th: 1435 days (95% CI: 342–2528) vs. 33rd-66th: 473 days (95% CI: 193–753) vs. <33rd: 403 days (95% CI: 0–828); log rank X^2^(2) = 9.324; *p* = 0.009; Fig. 3b). Furthermore, patients under the 66th percentile or under the 33rd percentile had a comparably worse survival, indicating that the value of BMD for OS might not be linear and only relevant above a certain level (Fig. 3b). Finally, we used the *optimal cut-off finder* to establish an ideal cut-off value for BMD discriminating between survivors and non-survivors in our cohort [12]. The use of this optimal cut-off value (156.5 HU) – which turned out as almost identical to the value for the 66th percentile—further increased the prognostic potential of BMD (1435 days (95% CI: 342–2528) vs. 459 days (95% CI: 212–706); log rank X^2^(1) = 9.223; *p* = 0.002; Fig. 3c).

To further substantiate the prognostic power of BMD, we next performed univariate Cox-regression analyses of routinely measured tumor markers for hepatobiliary malignancies (CEA, AFP, Ca19-9) and standard laboratory markers (hemoglobin, leukocyte count, thrombocyte count, sodium, potassium, calcium, AST, bilirubin, GGT, AP, CRP, INR and aPTT), as well as routine clinical and pathological parameters (age, gender, height, weight, BMI). In this analysis, serum levels of calcium, AST, age and BMD were predictive factors for long-term survival (Table 2). Subsequently, we included parameters with a significant result in the univariate analysis into a multivariate Cox-regression analysis, adding CEA and CA19-9 as relevant tumor markers for BTC. Strikingly, this multivariate analysis revealed BMD as the only independent marker predicting long-term survival after BTC tumor resection (HR 0.983 (95% CI: 0.967–0.999); *p* = 0.038; Table 2), supporting its use as a prognostic marker in patients receiving surgery for BTC.

**Table 2:** Univariate and multivariate Cox-regression analysis for the prediction of postoperative overall survival.

Parameter	Univariate cox regression		Multivariate cox regression	
	*p* value	Hazard ratio (95% CI)	*p* value	Hazard ratio (95% CI)
Sex	0.266	0.730 (0.419–1.271)		
**Age**	**0.027**	**1.034 (1.004–1.064)**	0.320	0.968 (0.907–1.032)
Height cm	0.855	0.997 (0.971–1.025)		
Weight kg	0.213	0.990 (0.976–1.006)		
BMI	0.208	0.963 (0.909–1.021)		
Sodium	0.684	0.986 (0.922–1.054)		
Potassium	0.149	0.647 (0.358–1.169)		
Creatinin	0.407	0.787 (0.446–1.388)		
GFR ml/min	0.484	0.996 (0.985–1.007)		
Urea	0.710	1.004 (0.981–1.028)		
Uric acid	0.920	1.002 (0.960–1.046)		
Bilirubin	0.191	1.116 (0.947–1.316)		
γGT	0.297	1.000 (1.000–1.001)		
AP	0.195	1.001 (1.000–1.002)		
Albumin	0.569	0.803 (0.377–1.710)		
**CRP**	**0.001**	**1.112 (1.043–1.186)**	0.155	1.101 (0.964–1.257)
TSH	0.885	0.986 (0.811–1.198)		
**Calcium**	**0.005**	**0.162 (0.046–0.572)**	0.827	0.758 (0.064–9.029)
**CEA**	**0.112**	**1.014 (0.997–1.031)**	0.124	1.015 (0.996–1.034)
AFP	0.217	1.017 (0.990–1.044)		
**CA19-9**	**0.069**	**1.000 (1.000–1.000)**	0.722	1.000 (1.000–1.000)
INR	0.230	3.833 (0.427–34.423)		
aPTT	0.160	1.032 (0.988–1.078)		
**Bone mineral density L1**	**0.031**	0.992 (0.985–0.999)	0.038	0.983 (0.967–0.999)

*BMI* body-mass-index, *GFR* glomerular filtration rate, *AST* aspartate-aminotransferase, *γGT* γ-glutamyltransferase; *AP* alkaline phosphatase, *CRP* C-reactive protein, *TSH* thyroid-stimulating hormone, *CEA* carcinoembryonic antigen, *AFP* α-fetoprotein, *CA19-9* carbohydrate antigen 19-9, *INR* International normalized ratio, *aPTT* activated partial thromboplastin time.

### Validation of the prognostic value of BMD in an external cohort

**Fig. 4:**
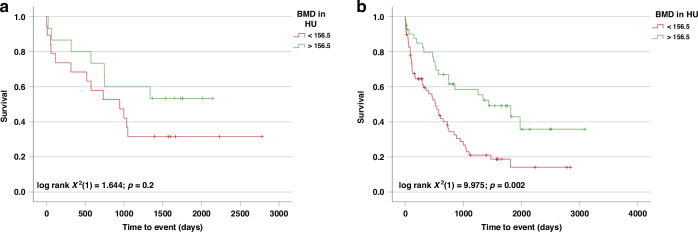
BMD as a prognostic marker in the validation (Cologne) and combined cohort. Using the established optimal cut-off value for BMD from our Duesseldorf cohort (156.5 HU), a strong trend towards improved survival was found in the external validation cohort (Cologne cohort) (**a**). Combining both cohorts (combined cohort), BMD above 156.5 HU indicated statistically significant improved OS after resection of BTC (**b**).

For external validation, a cohort of 34 (20/34 events = documented deaths) patients receiving curative intended liver surgery for BTC at the University Hospital Cologne was analyzed. Statistical significance was closely missed. This could be related to the small sample size of our validation cohort, but also other factors could impact this finding. Yet, patients with BMD values above the previously identified optimal cut off of 156.5 HU demonstrated a longer survival than those with lower BMD values (log rank X2(1) = 1.644; *p* = 0.2; Fig. 4a). To increase patient numbers, we combined both cohorts into a total cohort (combined cohort). In this analysis, patients with BMD values above 156.5 HU had a significantly improved OS (1435 days (95% CI: 699–2171) vs. 520 days (95% CI: 345–695); log rank X2(1) = 9.975, *p* = 0.002; Fig. 4b), confirming our initial finding that BMD is relevant for estimating the prognosis of patients receiving surgery for BTC.

### BMD of the first lumbar vertebra is a sex specific predictor for overall survival in patients undergoing surgery for BTC

**Fig. 5:**
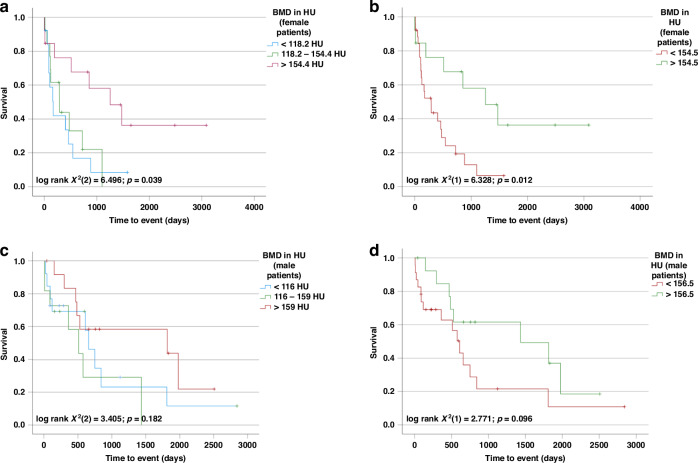
BMD is a prognostic marker in female, but not male patients after resection of BTC. Using the sex-specific tertiles (33rd and 66th percentile) in the Duesseldorf cohort, only female patients with high BMD had improved OS (**a**, **c**). Even after establishing sex-specific optimal cut-off values (female: 154.5 HU; male: 156.5 HU), only female patients with BMD above the cut off had improved OS (**b**), while in male patients no statistical significance was observed (**d**).

We recently demonstrated that the BMD is a predictor of mortality in female (but not in male) patients with BTC undergoing systemic chemotherapy, suggesting that the prognostic role of markers of body composition might be sex specific [13]. To test whether this observation might also hold true in patients receiving surgery, we performed Kaplan–Meier analysis separately in male and female patients of the Duesseldorf cohort. Using sex-specific tertiles only female (but not male) patients with BMD above the 66th percentile had a longer OS compared to patients with a BMD below this threshold (female: 1254 days (95% CI: 417–2091) vs. 290 days (95% CI: 26–554) vs. 172 days (95% CI: 62–282); log-rank X2(2) = 6.492; *p* = 0.039; male: 1814 days (95% CI: 0–4674) vs. 511 days (95% CI: 148–874) vs. 657 days (95% CI: 468–846); log-rank X2(2) = 3.405; *p* = 0.182; Fig. 5a, c). Interestingly, again a certain BMD threshold seems to be relevant for having an impact on OS as in female patients both curves of patients under the 33rd percentile but also between 33rd and 66th percentile seem to run in parallel. To further investigate potential differences between males and females, we used the optimal cut-off finder and determined sex-specific optimal cut-off values for BMD at L1. Within females a BMD value of 154.5 HU which is nearly identical with the sex-specific 66th percentile (154.4 HU), best discriminated between survivors and non survivors (1254 days (95% CI: 417–2091) days vs. 289 days (95% CI: 92–486); log-rank X2(1) = 6.328; *p* = 0.012; Fig. 5b). In contrast, male patients with BMD values above or below 156.5 HU (proposed by the cut-off finder as optimal value) displayed similar survival times (1435 days (95% CI: 0–3015) days vs. 608 days (95%CI: 438–778); log-rank X2(1) = 2.771; *p* = 0.096; Fig. 5d). Next, we performed univariate Cox regression analysis separately in male and female patients. We confirmed the prognostic value of BMD in L1 for female patients but could not detect an impact in male patients (female: HR 0.989 (95% CI: 0.979–1.000); *p* = 0.046; male: HR 0.996 (95% CI: 0.987–1.005); *p* = 0.391) (Supplementary Table 2).

## Discussion

BTC is a rare but aggressive type of cancer. Despite various multimodal treatment options, including chemotherapy and surgery, survival rates of patients with BTC have not substantially improved in recent years [4]. With 5-year survival rates of only 7–20%, BTC is one of the most aggressive and treatment-resistant cancers, having the second-worst prognosis after pancreatic cancer. There is urgent need for improved clinical management of these patients, e.g. by identifying reliable and easily available markers for treatment selection [4]. Dual X-ray absorptiometry measurement (DXA) is the most reliable method for measuring BMD and is useful for assessing fracture risk and monitoring therapy for osteopenia [14–16]. However, it is not necessary for staging examinations in tumor patients and therefore not routinely performed. In contrast, CT scans are routinely performed before surgery in tumor patients. Since in most cases they can also be used to quantify BMD by determining radiodensity, the broad availability of CT scans eliminates the need for additional DXA for evaluating the patients´ body composition. Of note, recent studies have confirmed the accuracy of routine CT scans in assessing bone mineral density [8, 9], leading us to use this technique for our study. Surgical therapy cannot only alleviate symptoms, but also lead to long-term survival [17]. According to international guidelines, tumor resection is always recommended when complete resection (R0) is considered possible [17]. Yet just ~25% of BTC patients are deemed resectable at time of diagnosis [4]. In these patients, 5-year survival rates of 25–40% are achieved, depending on the stage of disease and patient selection [18]. The challenging decision of whether to perform surgery or recommend a more conservative therapy is often only based on the patient’s performance status and the technical feasibility of surgery, rather than on specific and objective indicators. Prognostic biomarkers are needed for the clinical management of a patient and used as decision aids to plan therapeutic approaches and should be reported according to the REMARK guidelines [19]. Biomarkers that can be measured before surgery may help to better identify patients who might benefit from additional attention in matters of prehabilitation before tumor surgery as part of a personalized treatment plan [20]. However, current tumor markers including the well-established CEA and CA19-9 often fail to identify the best candidates for surgery, although having generally proven useful in diagnosis, resection evaluation or treatment monitoring [4, 21]. Here, we demonstrate in two independent cohorts of patients with BTC receiving curative-intend surgical tumor resection that BMD as a prognostic marker provides valuable information on the patients’ postoperative outcome, as patients with impaired BMD had a significantly worse overall survival compared to other patients. Of note, our results are in line with previous findings in pancreatic cancer or colorectal cancer [22, 23]. In our analyses, BMD levels below a calculated optimal cut-off value (156.5 HU) identified a high-risk subgroup of BTC patients showing a substantially impaired long-term prognosis with a median OS of only 459 days. The prognostic relevance of BMD was further corroborated by uni- and multivariate Cox-regression analyses including various clinical and pathophysiological confounders. BMD is a measure of the amount of mineral, primarily calcium, in bones. Reduction of BMD is an increasingly important phenomenon, especially in an aging society, and the medical consequences are profound. It has already been shown that low BMD is associated with various conditions and diseases like cardiovascular diseases, stroke or chronic lung disease [24–26]. In oncology, an impaired BMD was recently identified as an independent marker for a poor prognosis in patients with digestive tract cancer [7] or in patients receiving palliative chemotherapy for BTC [13]. Our data on the prognostic role of BMD in patients receiving surgery for BTC therefore nicely integrate in the available body of literature and further support the broad use of such markers in the preoperative stratification of cancer patients. Nevertheless, it is unlikely that a patient who is a surgical candidate based on their performance status and imaging results would be denied surgery due to a single marker such the preoperative BMD. We therefore propose that preoperative measurements of BMD may be useful in identifying a group of high-risk BTC patients who should be closely monitored, especially in terms of clinical measurements related to cachexia or organ dysfunction. This could lead to more aggressive perioperative treatment for high-risk BTC patients or strategies to alleviate these findings in a prehabilitation setting. While clinical trials are currently investigating more aggressive adjuvant chemotherapy regimens, biomarkers such as BMD may help to identify patients who would particularly benefit from perioperative treatment or prehabilitation in terms of improved survival and/or quality of life.

We provide evidence that the prognostic value of BMD measurements is more prominent in women than in men, suggesting a sex-specific role of BMD in stratifying patients for surgery or other treatment modalities. These finding are striking since they reproduce our previous data from BTC-patients receiving systemic chemotherapy [13]. Moreover, they are in line with previous data providing evidence for muscle mass—another marker of the patients´ body composition - as a sex-specific prognostic factor in patients receiving surgery for BTC, where sarcopenia is only relevant in male but not in female patients [5]. Sarcopenia has also been demonstrated as relevant prognostic factor after liver transplantation only in male, but not in female patients [27]. The differences in BMD between men and women can be attributed to specific hormonal changes. BMD is considered an indicator of lifetime exposure to endogenous estradiol, a female hormone [28]. In women, the levels of reproductive hormones decline to prepubertal levels during midlife, whereas in men, they remain relatively stable until advanced age [29, 30]. Moreover, a substantial decrease in gonadal hormone levels is required to significantly affect BMD [31]. Thus, changes in estradiol levels strongly impact BMD in women [32], which may explain the observed effects. Testosterone, the primary male hormone, has a particularly strong influence on muscle mass [33]. Additionally, estradiol in men is primarily produced by the conversion of testosterone, and the levels of gonadal hormones are closely linked [34, 35]. The sex-specific changes in gonadal hormone levels may account for the differential changes in BMD and muscle mass between men and women. These findings also shed light on why BMD might be more relevant for women, whereas muscle mass is more relevant for men.

Our study has some limitations, which currently prevent applying its results to patients in clinical routine. First, our study included only 110 patients, representing a rather small cohort of patients when analyzing complex endpoints, such as OS. Second, our study represents a retrospective analysis conducted at two centers only, thus center-specific bias cannot be excluded. Furthermore, our validation cohort only consists of 34 patients due to the rarity of BTC. Moreover, due to the rarity of BTC, patients were included over a very long period of time, during which the methodology of liver surgery and postoperative treatment has changed. In addition, patients with differing demographics, risk factors, tumor localization, molecular structures and treatment approaches were included. Therefore, it cannot be ruled out that these effects may have impacted the results of the study. However, and most importantly, our study only looked at surgical resection as a treatment approach for BTC and did not include alternative treatments such as chemotherapy or loco-regional therapies. Thus, we cannot determine if patients with an initial BMD below our cut-off value would have had better outcomes with different treatments. Therefore, further research with larger patient numbers and multiple treatment modalities is needed to fully understand the relationship between BMD measurements and BTC surgery outcomes and potentially influence specific prehabilitation measurements before BTC surgery.

## Supplementary information

Supplementary material

## Data Availability

Data included in this analysis represent highly sensitive medical data. It is directly against German (and European) law to publish such data in a way that would allow individual patients to be identified (e.g., by providing different clinical values of one distinct patient). Data are available upon request from the Department of Gastroenterology, Hepatology and Infectious Diseases at the University Hospital Düsseldorf for researchers who meet the criteria for access to confidential data: Wissenschaft.Gastro@med.uni-duesseldorf.de.
