# Supplementary material for: Bone mineral density as a prognostic marker in patients with biliary tract cancer undergoing surgery
Source: BJC Rep. 2024 Sep 23;2:72. doi: 10.1038/s44276-024-00094-2 (PMC11420066; doi:10.1038/s44276-024-00094-2)
Supplement: Supplementary file 1 — Supplementary material [file 44276_2024_94_MOESM1_ESM.docx]

**Supplemental material**

**Supplemental Figure 1:**

BMD assessment at first lumbar vertebra using CT in venous phase and local PACS (IntelliSpace PACS, Philips, Amsterdam, The Netherlands). Examples for high (A) and low (B) BMD values.

**Supplemental Table 1: Study Cohort Cologne**

| Parameter | Study Cohort Cologne |
| --- | --- |
| BTC patients | n = 34 |
| Sex (%) |  |
| male | 61.8 (21) |
| female | 38.2 (13) |
| Age (years, median and range) | 68 (44 – 90) |
| Tumor localization (%) |  |
| iCCA | 82.4 (28) |
| eCCA | 17.6 (6) |
| Chemotherapy (%) |  |
| adjuvant | 24 (70.59) |
| Neoadjuvant | 4 (11.76) |
| Staging (%) |  |
| UICC I | 17.6 (6) |
| UICC II | 41.2 (14) |
| UICC III | 32.4 (11) |
| UICC IV | 8.8 (3) |
| Time to event (days, median and range) | 999 (3 – 2780) |
| BMD L1 (HU, median and range) | 140 (59.50 – 266.00) |

BTC: biliary tract cancer, iCCA: Intrahepatic cholangiocellular adenocarcinoma, eCCA: extrahepatic cholangiocellular adenocarcinoma, UICC: Union for International Cancer Control

**Supplemental Table 2: Univariate Cox-regression analysis for the prediction of sex-specific postoperative overall survival - Duesseldorf cohort**

|  | | **Univariate Cox Regression** | |
| --- | --- | --- | --- |
| **Parameter** | **Sex** | **p-value** | **Hazard Ratio (95% CI)** |
| Age | m | 0.288 | 1.023 (0.981 – 1.068) |
|  | f | 0.087 | 1.035 (0.995 – 1.078) |
| Height cm | m | 0.673 | 0.989 (0.937 – 1.043) |
|  | f | 0.172 | 1.035 (0.985 – 1.088) |
| Weight kg | m | 0.094 | 0.977 (0.951 – 1.004) |
|  | f | 0.384 | 1.013 (0.984 – 1.043) |
| BMI | m | 0.132 | 0.932 (0.851 – 1.021) |
|  | f | 0.683 | 1.019 (0.930 – 1.116) |
| Sodium | m | 0.059 | 0.887 (0.783 – 1.005) |
|  | f | 0.381 | 1.042 (0.950 – 1.144) |
| Potassium | m | 0.156 | 0.477 (0.172 – 1.326) |
|  | f | 0.360 | 0.716 (0.349 – 1.466) |
| Creatinin | m | 0.420 | 1.877 (0.407 – 8.660) |
|  | f | 0.324 | 0.720 (0.375 – 1.384) |
| GFR | m | 0.174 | 0.986 (0.965 – 1.006) |
|  | f | 0.993 | 1.000 (0.987 – 1.013) |
| Urea | m | 0.118 | 1.037 (0.991 – 1.085) |
|  | f | 0.816 | 0.997 (0.971 – 1.023) |
| Uric acid | f | 0.538 | 0.772 (0.338 – 1.761) |
|  | f | 0.856 | 1.004 (0.963 – 1.047) |
| Bilirubin | m | **0.002** | **3.925 (1.676 – 9.190)** |
|  | f | 0.975 | 0.997 (0.810 – 1.227) |
| AST | m | 0.879 | 1.001 (0.993 – 1.008) |
|  | f | **0.028** | **1.000 (1.000 – 1.001)** |
| γGT | m | 0.135 | 1.003 (0.999 – 1.006) |
|  | f | 0.604 | 1.000 (0.999 – 1.001) |
| AP | m | **0.003** | **1.011 (1.004 – 1.018)** |
|  | f | 0.721 | 1.000 (0.999 – 1.002) |
| Albumin | m | 0.133 | 0.153 (0.013 – 1.774) |
|  | f | 0.836 | 0.916 (0.399 – 2.102) |
| CRP | m | **0.009** | **1.127 (1.030 – 1.232)** |
|  | f | **0.094** | **1.087 (0.968 – 1.198)** |
| TSH | m | 0.618 | 0.904 (0.608 – 1.345) |
|  | f | 0.899 | 0.984 (0.770 – 1.257) |
| CEA | m | 0.262 | 1.048 (0.965 – 1.139) |
|  | f | 0.235 | 1.011 (0.993 – 1.029) |
| AFP | m | 0.342 | 1.021 (0.978 – 1.065) |
|  | f | 0.131 | 1.027 (0.992 – 1.064) |
| CA19-9 | m | 0.061 | 1.000 (1.000 – 1.000) |
|  | f | 0.958 | 1.000 (1.000 – 1.000) |
| INR | m | 0.699 | 2.320 (0.033 – 165.530) |
|  | f | 0.347 | 3.153 (0.287 – 34.599) |
| aPTT | m | 0.341 | 0.931 (0.802 – 1.079) |
|  | f | 0.115 | 1.035 (0.992 – 1.081) |
| Bone mineral density L1 | m | 0.391 | 0.996 (0.987-1.005) |
|  | **f** | **0.046** | **0.989 (0.979 – 1.000)** |

BMI: Body-Mass-Index, GFR: glomerular filtration rate, AST: aspartate-aminotransferase, γGT: γ-glutamyltransferase; AP: Alkaline phosphatase; CRP: C-reactive protein; TSH: Thyroid-stimulating hormone, CEA: Carcinoembryonic antigen, AFP: α-fetoprotein, CA19-9: Carbohydrate antigen 19-9, INR: International normalized ratio, aPTT: activated partial thromboplastin time, m: male, f: female
